# Thermostability enhancement of the α-carbonic anhydrase from *Sulfurihydrogenibium yellowstonense* by using the anchoring-and-self-labelling-*protein-tag* system (ASL*^tag^*)

**DOI:** 10.1080/14756366.2019.1605991

**Published:** 2019-04-30

**Authors:** Sonia Del Prete, Rosa Merlo, Anna Valenti, Rosanna Mattossovich, Mosè Rossi, Vincenzo Carginale, Claudiu T. Supuran, Giuseppe Perugino, Clemente Capasso

**Affiliations:** a Department of Biology Agriculture and Food Sciences, Institute of Bioscience and BioResources – National Research Council of Italy, Naples, Italy;; b Neurofarba Department, University of Florence, Polo Scientifico, Sesto Fiorentino Firenze, Italy

**Keywords:** Carbonic anhydrase, thermostability, *Sulfurihydrogenibium yellowstonens;* alkylguanine-DNA-alkyl-transferase, ASL*tag*

## Abstract

Carbonic anhydrases (CAs, EC 4.2.1.1) are a superfamily of ubiquitous metalloenzymes present in all living organisms on the planet. They are classified into seven genetically distinct families and catalyse the hydration reaction of carbon dioxide to bicarbonate and protons, as well as the opposite reaction. CAs were proposed to be used for biotechnological applications, such as the post-combustion carbon capture processes. In this context, there is a great interest in searching CAs with robust chemical and physical properties. Here, we describe the enhancement of thermostability of the α-CA from *Sulfurihydrogenibium yellowstonense* (*Ss*pCA) by using the anchoring-and-self-labelling-*protein-tag* system (ASL*^tag^*). The anchored chimeric H^5^-*Ss*pCA was active for the CO_2_ hydration reaction and its thermostability increased when the cells were heated for a prolonged period at high temperatures (e.g. 70 °C). The ASL*^tag^* can be considered as a useful method for enhancing the thermostability of a protein useful for biotechnological applications, which often need harsh operating conditions.

## Graphical Abstract

**Figure:**
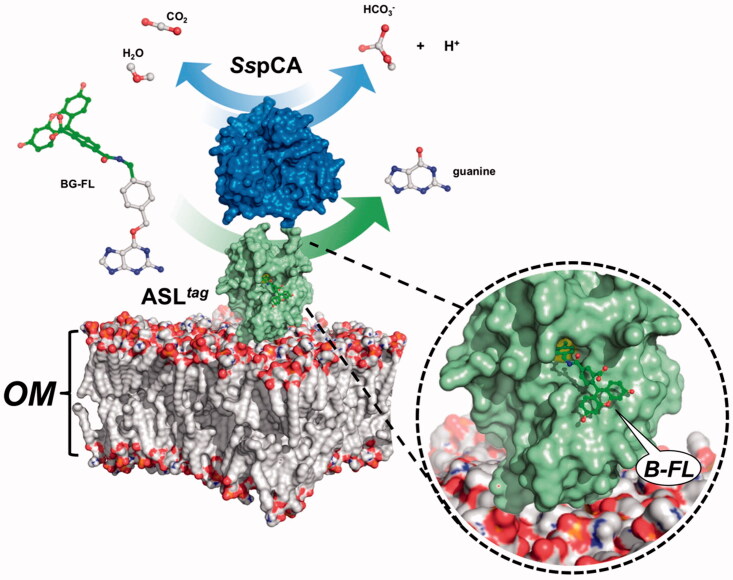

## 1. Introduction

The hydration/dehydration reaction involving carbon dioxide, water, bicarbonate, and protons (CO_2_ + H_2_O ⇄ HCO_3_^−^ + H^+^) is a fundamentally important process for the planet and all its associated forms of life^1–9^. The dissolution of CO_2_ in the aqueous phase develops carbonic acid (H_2_CO_2_), which is subject to an ionisation reaction producing bicarbonate (HCO_3_^−^), whereas this last species then generates carbonate (CO_2_) through a second dissociation reaction. These species are disseminated in the fluids of the all living organisms and are involved in a large number of physiological processes, such as some biosynthetic pathways, photosynthesis, respiration, pH homeostasis, secretion of electrolytes, etc.^9–11^. At physiological pH, the naturally uncatalysed CO_2_ hydration reaction has a catalytic constant (*k*_cat_) of 0.15 s^−1^, whereas the uncatalysed dehydration shows a *k*_cat_ of 50.0 s^−1^ ^12^^,^^13^. These values are typical of slow reactions and are not sufficient for accomplishing fast cellular physiological processes which support metabolic activities dependent on the dissolved inorganic carbon species (CO_2_, HCO_3_^−^, CO_3_^2−^)^9^. Probably this is the reason why living organisms evolved a superfamily of ubiquitous metalloenzymes, the carbonic anhydrases (CAs, EC 4.2.1.1), which catalyse, and highly accelerate, the above-mentioned reactions, at a very high rate with respect to the non-catalysed reaction. CAs show kinetic constants *k*_cat_ varying from 10^4^ to 10^6^ s^−1^ for the hydration reaction^12^^,^^13^.

Up to date, CA superfamily contains seven genetically distinct families (or classes), named α-, β-, γ-, δ-, ζ-, η-, and ɵ-CAs^9^^,^^14^^,^^15^, characterised by multiple transcript variants and protein isoforms, with different biochemical properties and specific tissue/organ and sub-cellular localisations^7^^,^^9^^,^^12^^,^^16–19^. Generally, only α-class enzymes are present in the animals^20^^,^^21^, whereas α-, β-, γ-, δ- and *θ*-classes are found in plants and algae; α- and β-CAs in fungi; α-, β-, and/or η-CAs in protozoans; α-, β-, and γ-CA classes in bacteria^7^^,^^19^^,^^15^^,^^22–25^.

Studies carried out on the bacterial CAs concern two main aspects. They are considered an attractive and rather new drug target, because their inhibition affects the growth or virulence of many pathogens^4^^,^^7^^,^^26–28^. Furthermore, they are biocatalysts often used in biotechnological applications^29^^,^^30^, such as the post-combustion carbon capture process, artificial lungs, and biosensors^31^^,^^32^. Many such processes are characterised by conditions, which may be deleterious to an enzyme belonging to the mesophilic organisms^25^^,^^33–45^. In the field of biotechnology, there is a great interest in searching proteins with robust chemical and physical properties, which resist the hard conditions of industrial processes. In this context, our groups identified in the genome of the extreme thermophiles *Sulfurihydrogenibium yellowstonense* and *Sulfurihydrogenibium azorense* two CAs, indicated with the acronyms *Ss*pCA and *Sa*zCA, respectively. It has been demonstrated that these two CAs belong to the α-CA class and showed an excellent activity as catalysts for the CO_2_ hydratase reaction (*k*_cat _=10^5^–10^6^ s^−1^)^30^^,^^46–52^. Interestingly, the two extreme enzymes resulted to be highly thermostable, retaining an excellent catalytic activity when heated for a prolonged period at a temperature higher than 80 °C^30^^,^^46–52^. The X-ray tridimensional structures of the two proteins demonstrated that the high compactness of the dimeric structure, the higher content of secondary-structural elements, the increased number of charged residues on the protein surface, and the vast number of ionic networks with respect to the mesophilic counterparts, are the main structural elements responsible for the protein thermostability^29^^,^^30^. Moreover, Russo et al. reported the use of free *Ss*pCA in experiment of CO_2_ absorption^53^ demonstrating that it is an excellent candidate for the biomimetic capture of CO_2_. Subsequently, the necessity to use this biocatalyst repeatedly and continuously, led to the immobilisation of the recombinant *Ss*pCA on polyurethane foam (PU), a pre-polymer of polyethylene glycol^54^; onto supported ionic liquid membranes (SMLs), in order to realise a system able to selectively separate and transform CO_2_^55^. Furthermore, the immobilisation onto magnetic support for recovering the biocatalyst from the bioreactor effortlessly and practically, for example through the use of a magnet, was also proposed for these thermostable CAs^56^. Unfortunately, these strategies may discourage the wide utilisation of enzymes in industrial applications because of the high costs connected to the biocatalyst production and purification, and the expenses for the preparation of the immobilisation support. Thus, to overcome this limitation, *a one-step immobilisation procedure* has been proposed, which consists in the overexpression of *Ss*pCA directly onto the surface of bacterial hosts, by using the ice nucleation protein (INP) from the Gram-negative bacterium *Pseudomonas syringae*^57^.

In this article, we describe the improvement of the thermostability of *Ss*pCA by using a novel *protein-tag* system, the ASL*^tag^*^58^. The anchored *Ss*pCA was fused to the thermostable variant of the alkylguanine-DNA-alkyl-transferase (H^5^) from the hyperthermophilic archaeon *Sulfolobus solfataricus*. The chimeric H^5^-SspCA was efficiently overexpressed on the bacterial surface of *Escherichia coli*. The protonography technique showed that the neosynthetised H^5^-*Ss*pCA was active for the CO_2_ hydration reaction. Even more intriguing, the chimeric H^5^-*Ss*pCA expressed onto the bacterial surface resulted to be more stable with respect to the non-chimeric *Ss*pCA, when treated at high temperatures (50.0 and 70.0 °C) for a prolonged time. The ASL*^tag^* system may thus be considered as a brilliant strategy to further increase the thermostability of proteins to be used in biotechnological applications, in which a highly effective and thermostable catalyst is needed.

## 2. Materials and methods

### 2.1 Construction of vectors for surface fusion and H^5^-SspCA overexpression

The vector pET-22b/INPN-*Ss*pCA was used to produce the pET-ASL*^tag^*-*Ss*pCA vector, which overexpressed onto the bacterial surface the chimeric H^5^-*Ss*pCA. The pET-22b/INPN-*Ss*pCA and pET-ASL*^tag^*-*Ss*pCA vectors were prepared as described previously^57^^,^^58^. For overexpressing the chimeric H^5^-*Ss*pCA or *Ss*pCA on the bacterial cell surface, competent *E. coli* BL21 (DE3) cells were transformed with the above-mentioned constructs. They were grown at 37.0 °C and induced with 1.0 mM isopropyl-thio-β-D-galactoside (IPTG) and 0.5 mM ZnSO_4_ at an OD_600_ of 0.6–0.7. After additional growth for 6 h, the cells were harvested by centrifugation and washed three times with PBS 1×. Aliquots of cells were resuspended in 25 mM Tris/HCl and used to determine the enzyme activity and for the preparation of the outer membrane fraction.

### 2.2 Carbonic anhydrase assay and SDS-PAGE

CA activity assay was a modification of the procedure described by Capasso et al.^59^. Briefly, the assay was performed at 0 °C using CO_2_ as substrate and following the pH variation due to the catalysed conversion of CO_2_ to bicarbonate. Bromothymol blue was used as the indicator of pH variation. The production of hydrogen ions during the CO_2_ hydration reaction lowers the pH of the solution until the colour transition point of the dye is reached. The time required for the colour change is inversely related to the quantity of CA present in the sample. Wilbur-Anderson units (WAU) were calculated according to the following definition: one WAU of activity is defined as (T_0_−T)/T, where T_0_ (uncatalysed reaction) and T (catalysed reaction) are recorded as the time (in seconds) required for the pH to drop from 8.3 to the transition point of the dye in a control buffer and in the presence of enzyme, respectively. Assay of the membrane-bound enzyme (H^5^-*Ss*pCA or *Ss*pCA) was carried out using an amount of whole cells or outer membranes ranging from 1.0 to 5.0 mg. Sodium dodecyl sulphate (SDS)-polyacrylamide gel electrophoresis (PAGE) was performed as described by Laemmli using 12% gels.^60^ Samples were dissolved in buffer with 5% β-mercaptoethanol. The gel was stained with Coomassie blue and protein concentration was determined by Bio-Rad assay kit (Bio-Rad, Hercules, CA).

### 2.3 Protonography and his-tag Western blotting

To perform the protonography, wells of 12% SDS-gel were loaded with solubilised outer membranes having on their surface H^5^-*Ss*pCA or *Ss*pCA, and a solution of free *Ss*pCA (enzyme overexpressed as cytoplasmic protein and purified as described previously^59^). Samples were mixed with loading buffer without 2-mercaptoethanol and without boiling the samples, to solubilise cells and avoid protein denaturation. The gel was run at 180 V until the dye front moved off the gel. Following the electrophoresis, the 12% SDS-gel was subject to protonography to detect the cytoplasmic *Ss*pCA, the surface-*Ss*pCA, and surface-H^5^-*Ss*pCA hydratase activity on the gel as described by Del Prete et al.^61^^,^^62^ and De Luca et al.^63^. To perform the Western-Blot, after a 12% (w/v) SDS-PAGE, the overexpressed cytoplasmic *Ss*pCA and the membrane-bound enzymes (*Ss*pCA and H^5^-*Ss*pCA) were also electrophoretic transferred to a PVDF membrane with transfer buffer (25 mM Tris, solubilised whole cells 192 mM glycine, 20% methanol) by using Trans-Plot SD Cell (Bio-Rad, Hercules, CA). His-tag Western blot was carried out using the Pierce Fast Western Blot Kit (Thermo Scientific, Waltham, MA). The blotted membrane has been placed in the wash blot solution Fast Western 1× Wash Buffer to remove transfer buffer. Primary Antibody Working Dilution was added to the blot and incubated for 30.0 min at room temperature (RT) with shaking. After, the blot was removed from the primary antibody solution and incubated for 10.0 min with the Fast Western Optimized HRP Reagent Working Dilution. Subsequently, the membrane was washed two times in about 20 ml of Fast Western 1× Wash Buffer. Finally, the membrane was incubated with the Detection Reagent Working Solution and incubated for 3.0 min at RT and then developed with X-ray film.

### 2.4 Determination of the H^5^ activity by an in vitro and in vivo fluorescent assay

Whole overnight inducted *E. coli* BL21(DE3) cells were collected and the expression of the H^5^-derived fusion proteins was analysed by an *in vitro* assay with the fluorescent SNAP-Vista Green™ substrate (New England Biolabs, Ipswich, MA; hereinafter BG-FL), as previously described^58^^,^^64^. The *in vivo* imaging was carried out as described by Merlo et al.^58^. Briefly, bacterial cells expressing the H^5^-*Ss*pCA onto cell surface were washed twice in PBS 1× and resuspended in 50.0 μl of the same buffer supplemented with 5.0 μM of the BG-FL. After incubation at 37.0 °C for 30.0 min, cells were washed twice, resuspended, and again incubated for 30.0 min at 37.0 °C, to allow the external diffusion of the unreacted substrate. Images were collected using a DM6 fluorescence microscope and Hamamatsu camera under the control of Leica LAS AF 6000 software; excitation and emission wavelengths used suitably for AlexaFluor488 dye were λ_ex_ = 490 nm; λ_em_ = 525 nm, respectively.

### 2.5 Outer membrane preparation

The bacterial outer membranes were fractioned by inner membranes as described previously by Del Prete et al.^57^. Briefly, 2.0 g of harvested bacterial cells were resuspended and disrupted by sonication on the ice. Cell extract was ultracentrifuged to recover the total membrane fraction. The outer membrane fraction was purified resuspending the pellet in phosphate-buffered saline (PBS 1×) containing 0.01 mM MgCl_2_ and 2% Triton X-100 and incubated at RT for 30.0 min to solubilise the inner membrane. The outer membrane fraction was then pelleted by ultracentrifugation at 120,000×*g* and used for further experiments.

### 2.6 Temperature stability studies

#### 2.6.1 Thermostability

Bacterial cells (2.0 g/20 ml) were incubated at 25.0, 50.0, and 70.0 °C for different time up to 24 h to compare the stability of the membrane-bound enzymes (*Ss*pCA and H^5^-*Ss*pCA) at the above-indicated temperatures. Cell membrane-bound enzymes aliquots were withdrawn at appropriate times and the residual activity was measured using CO_2_ as the substrate. All data have been analysed using GraphPad Prism version 5.0 software (GraphPad Software, San Diego, CA). Curves were obtained by the mean of three independent determinations.

#### 2.6.2 Long-term stability

Membrane-bound *Ss*pCA or H^5^-*Ss*pCA were investigated for their long-term stability at different temperatures (25.0, 50.0, and 70.0 °C) by assaying their hydratase residual activities using CO_2_ as substrate and withdrawing aliquots of cell surface *Ss*pCA or H^5^-*Ss*pCA at appropriate times. All the buffers were sterilised by using a sterile 0.22 μm filter, while samples containing the membrane-bound enzymes were treated with a diluted solution of NaN_3_ to avoid contamination. All data were obtained by the mean of 3 independent determinations.

## 3. Results and discussion

### 3.1 Expression and surface localisation of SspCA and H^5^-SspCA

**Figure 1.**
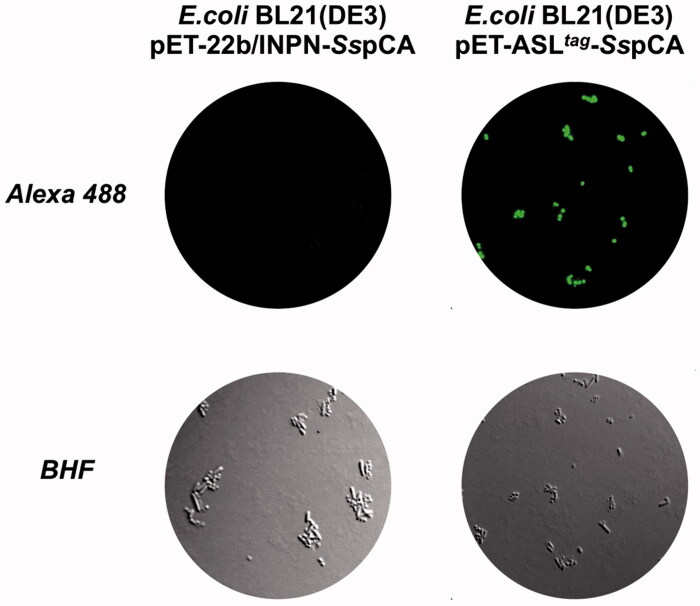
Fluorescence microscopy of *E. coli* BL21(DE3) cells transformed with pET-22b/INPN-*Ss*pCA (left) or with pET-ASL*^tag^*-*Ss*pCA (right). The cells were incubated with BG-FL and then analysed by fluorescence microscopy. Images show bright field (BHF) and AlexaFluor488 (green). As expected, the fluorescence is only evidenced for the bacterial cell transformed with the ASL*^tag^* system.

**Figure 2.**
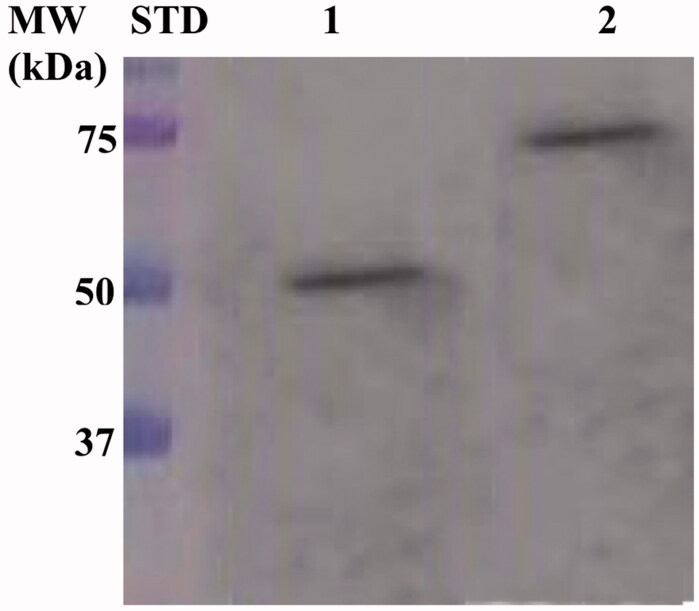
Western Blot performed on the outer membrane purified from the whole bacterial cells. The anti His-tag antibody was raised against the C-terminus of His-tagged *Ss*pCA. Legend: Lane Std, molecular markers, M.W. starting from the top: 75.0, 50.0, and 37.0 kDa; Lane 1, anchored *Ss*pCA; Lane 2, anchored H^5^-*Ss*pCA.

**Figure 3.**
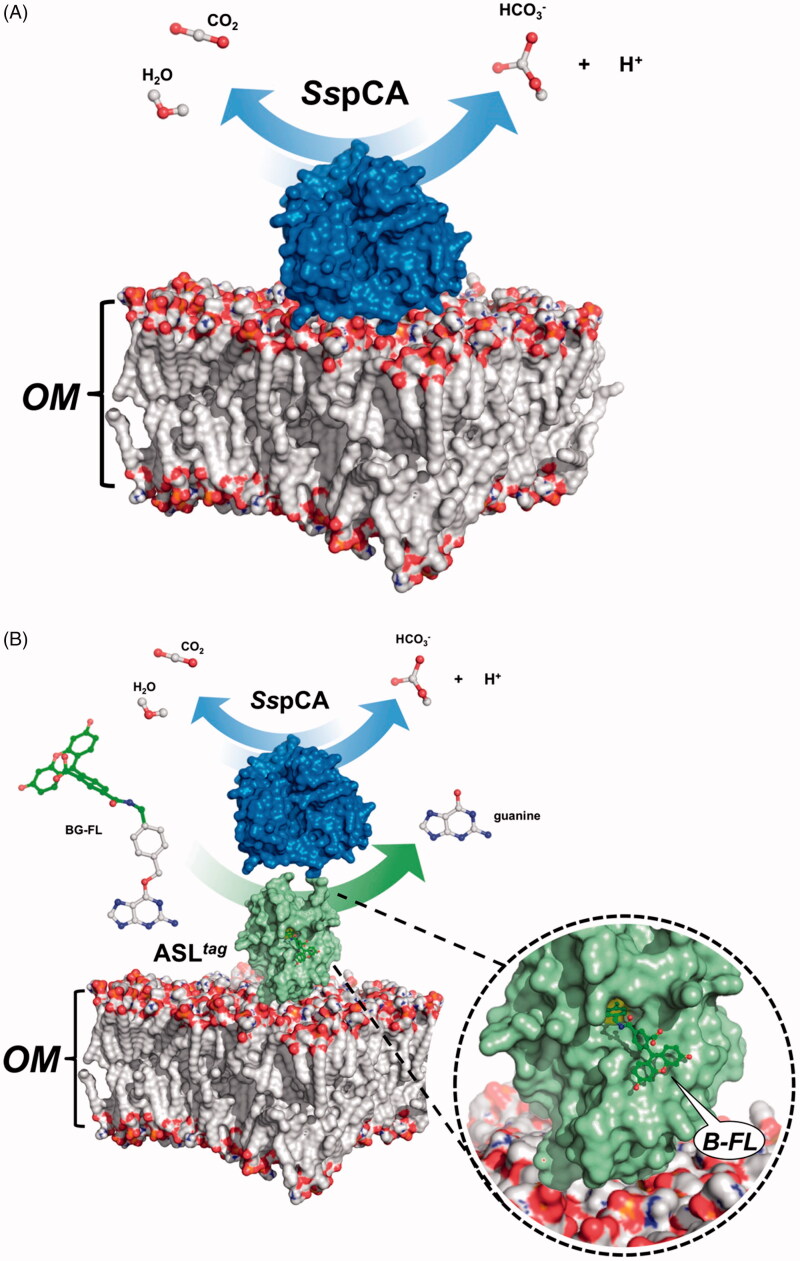
Model representation of an outer membrane fraction (OM; pdb from Tieleman and Berendsen^65^) describing the *in vivo* immobilisation of *Ss*pCA (*in blue*; PDB ID: 4G7A; panel A) and in fusion with H^5^ (*in green*; PDB ID: 6GA0; panel B). The INPN domain is omitted because inserted in the OM. The catalytic reaction of *Ss*pCA (the hydration/dehydration of CO_2_) and H^5^ (the conversion of BG-FL in the free guanine and the fluorescent benzyl-guanine derivative, B-FL, covalently linked to the active site of H^5^) are also shown.

Expression of the *Ss*pCA and H^5^-*Ss*pCA was realised through *the one-step procedure*, by transforming the *E. coli* cells with the construct expressing a gene composed of a signal peptide (necessary for the periplasmic translocation of the protein), the *P. syringae* INPN domain (fundamental for displaying the overexpressed protein onto the bacterial surface), and the protein of interest (*Ss*pCA or H^5^-*Ss*pCA). This strategy has the advantage to overexpress and directly immobilise *in vivo* the α-CA or other proteins on the bacterial cell surface. Besides, the system expressing the H^5^-*Ss*pCA, named anchoring-and-self-labelling-*protein-tag* (ASL*^tag^*), allowed the labelling of the neosynthesised protein fused to H^5^ through the use of the fluorescein derivative of the *O^6^*-BG (BG-FL), which is the substrate of H^5^. As reported in Figure 1, the expression of the chimeric H^5^-*Ss*pCA on the bacterial surface has been confirmed using the H^5^ substrate and analysing the whole bacterial cells with fluorescent microscopy. The irreversible reaction of the ASL*^tag^* system with a fluorescent substrate allowed the quantitative determination of the immobilised bacterial α-CA or of other proteins fused to H^5^, by *in vitro* gel-imaging techniques as described by Del Prete et al.^57^ and Merlo et al.^58^. Diversely from H^5^-*Ss*pCA, the expression of the anchored His-tagged *Ss*pCA (without the H^5^) has been confirmed only by the Western Blot analysis using an anti-His-tag antibody (Figure 2), indicating an expected molecular weight of 50.0 kDa (the sum of the INPN and *Ss*pCA polypeptide chains produced with the construct pET-22b/INPN-*Ss*pCA; see the experimental section). Anchored His-tagged H^5^-SspCA showed a higher molecular weight (70.0 kDa) with respect to the non-chimeric protein because of the presence of the H^5^ protein (158 amino acid residues). The H^5^-*Ss*pCA Western-Blot fully supports the fluorescence microscopy results. Thus, using this *one-step procedure*, the thermostable proteins α-CA (*Ss*pCA) and the chimeric ASL*^tag^* -*Ss*pCA^56^ were efficiently expressed on the external side of the bacterial outer membrane. Figure 3 reports a model representing the *in vivo* immobilisation of *Ss*pCA (Panel A) and chimeric H^5^-*Ss*pCA (Panel B) on the bacterial external cell surface. Moreover, Figure 3 shows the reactions catalysed by both the biocatalysts (the hydration/dehydration of CO_2_ and the conversion of BG-FL in the free guanine and the fluorescent benzyl-guanine derivative covalently linked to the active site of H^5^).

### 3.2 Hydratase activity of the membrane anchored SspCA and H^5^-SspCA

**Figure 4.**
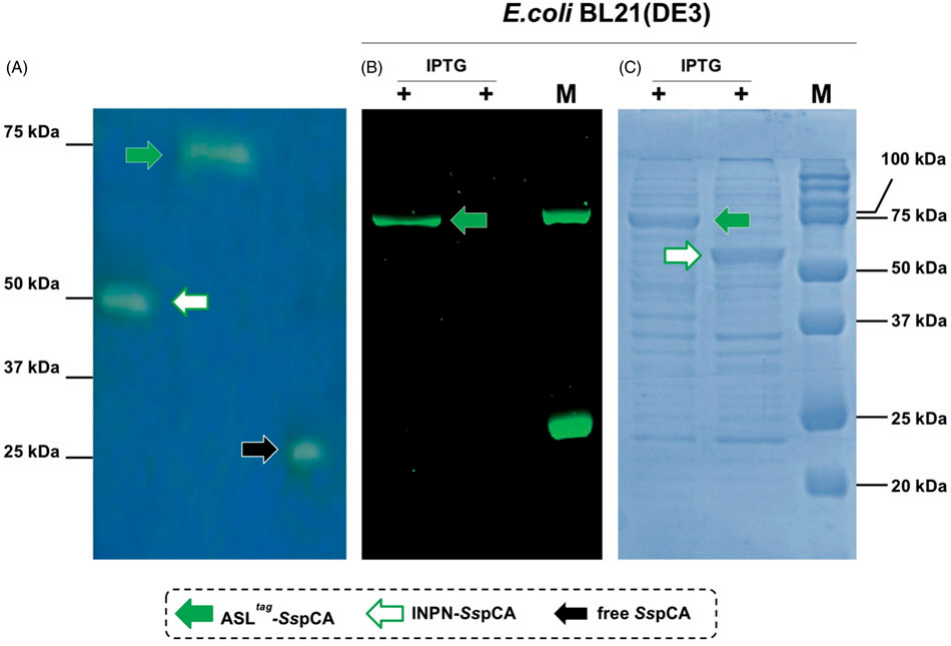
Protonography (Panel A), fluorescence *gel-imaging* (Panel B) and Coomassie staining (Panel C) of *Ss*pCA and H^5^-*Ss*pCA carried out with different amounts of the whole *E. coli* cells (see Materials and Methods). Filled green, white and black arrows represent the ASL*^tag^*-*Ss*pCA, INPN-*Ss*pCA and the free *Ss*pCA, respectively.

Using CO_2_ as the substrate, the hydratase activity of all the forms of *Ss*pCA has been investigated in solution^57^. The results showed that the membrane-bound *Ss*pCA with and without H^5^ was an active enzyme, when immobilised on the bacterial surface. The CO_2_ hydratase activity of *Ss*pCA and H^5^-*Ss*pCA did not show any differences. The results also evidenced that 1.0 µg of bacterial cells had a CO_2_ hydratase activity corresponding to that of 10.0 ng of free *Ss*pCA. Probably, anchored *Ss*pCA or H^5^-*Ss*pCA is subjected to various phenomena, which influence the enzymatic reaction, e.g. a reduction of the structural conformational changes (this is typical of an immobilised enzyme); a different substrate access to the active site with respect to the free biocatalyst due to the bacterial cell surface microenvironment, and, finally, an aggregation of the cells or outer membranes used in the assay. Otherwise, the activity of *Ss*pCAs was compared by using the protonography, which is a technique able to reveal the hydrogen ions produced by the hydratase activity reaction as a yellow band on the SDS-PAGE. The protonography results showed that the all the forms of *Ss*pCA (the two membrane-bound ones and the free enzyme) had a comparable enzyme activity and a different molecular weight on SDS-PAGE (Figure 4, panel A and C). Protonography corroborated the results obtained with the fluorescent microscopy (Figure 1) and Western Blot (Figure 2). Interestingly, H^5^-*Ss*pCA fluorescent band at a molecular weight of about 70.0 kDa (Figure 4, panel B) indicated that the presence of *Ss*pCA does not affect the activity of the thermostable H^5^ enzyme on the BG-FL substrate.

### 3.3 Stability of SspCA and H^5^-SspCA linked to the bacterial cell surface

**Figure 5.**
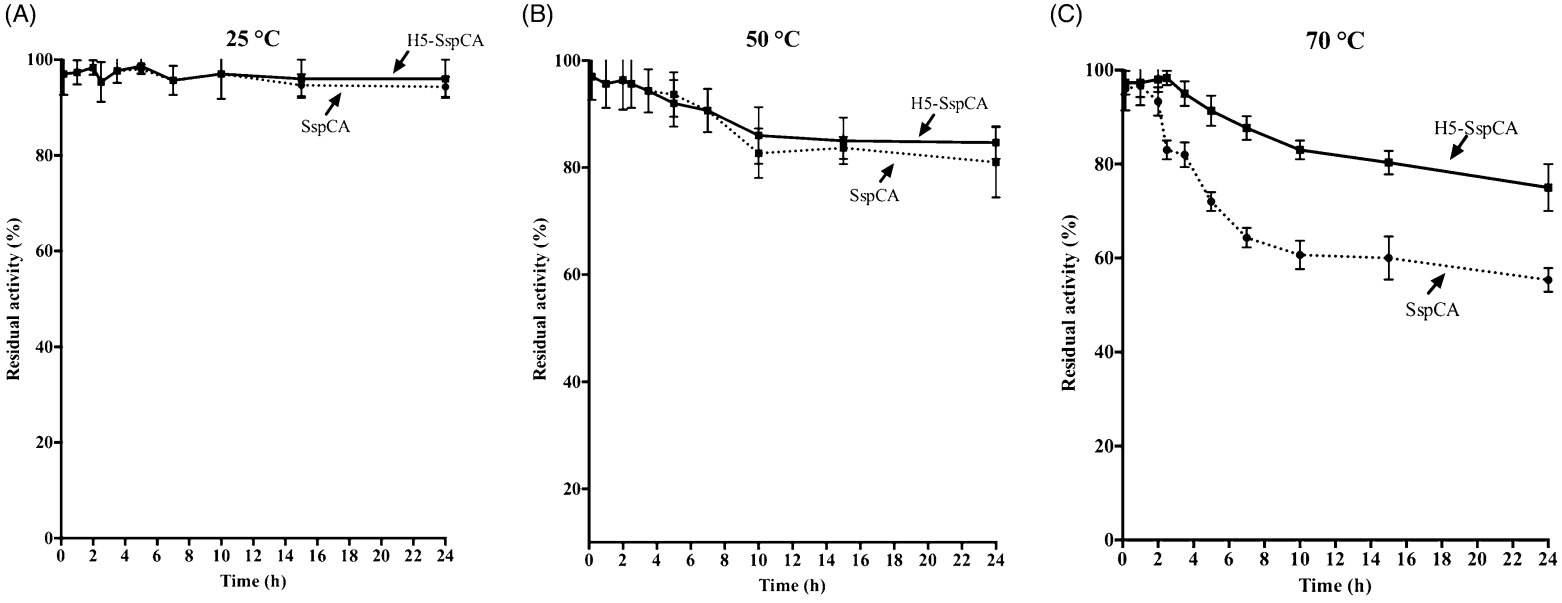
The thermostability of immobilised *Ss*pCA and H^5^-*Ss*pCA on the bacterial surface. Measures were carried out at indicated temperatures, by using aliquots of the whole cells incubated up to 24 h. Legend: continuous line, membrane-bound H^5^-*Ss*pCA; dashed line, membrane-bound *Ss*pCA. Each point is the mean of three independent determinations.

**Figure 6.**
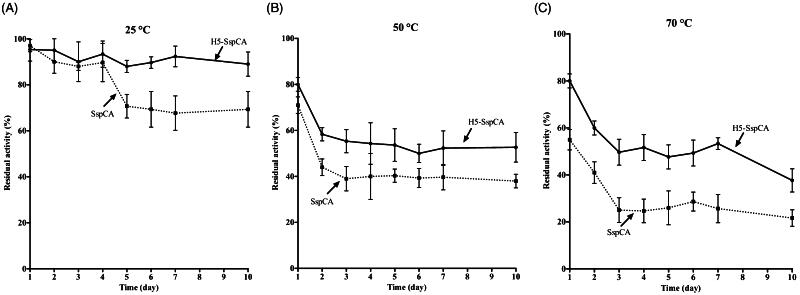
The long-term stability of immobilised *Ss*pCA and H^5^-*Ss*pCA on the bacterial surface. Measures were carried out at indicated temperatures up to 10 d, using aliquots of whole bacterial cells. Continuous line: free *Ss*pCA; Dashed line: membrane-bound *Ss*pCA. Each point is the mean of three independent determinations.

Using the whole bacterial cells expressing on the external surface *Ss*pCA or H^5^-*Ss*pCA, the effect of the CO_2_ hydratase reaction as a function of temperature has been investigated. In Figure 5, the residual activity of the *Ss*pCA and H^5^-*Ss*pCA remained almost constant at 25.0 and 50.0 °C, retaining their residual activity at 100% up to 24 h (panel A) and at 70% up to 6 h of incubation (panel B), respectively. In contrast, it is readily apparent that at higher temperatures (70.0 °C) *Ss*pCA and H^5^-*Ss*pCA behave differently (Figure 5, panel C): the residual activity of *Ss*pCA started to decline rapidly after 2 h, getting a value of about 60% after 14 h of incubation; whereas the stabilising effect of H^5^ on the *Ss*pCA showed a residual activity of about 85% and remained almost constant for the rest of the time indicated in the figure (panel C). These results demonstrated that the anchoring ASL*^tag^* system, enhanced the *Ss*pCA stability of about 20%. On the other hand, it is important to highlight that both anchored enzymes continued to work for several hours at temperatures considered prohibitive for the free enzymes, as *Ss*pCA, which Russo et al. demonstrated to show a residual activity of 20% when heated at 70.0 °C for 15 min^57^. This aspect is crucial in the context of the post-combustion carbon capture process, which requires temperatures ranging from 40.0 and 60.0 °C^53^. Figure 6 shows the residual activity for the CO_2_ hydration reaction for *Ss*pCA and H^5^-*Ss*pCA when the whole cells were treated at different temperatures for a very long period (up to 10 d). At 25.0 °C, the *Ss*pCA residual activity started to decrease after 4 d and reached a value of about 70% after 10 d, while H^5^-*Ss*pCA remained almost constant (panel A). At 50.0 and 70.0 °C, the residual activity of *Ss*pCA decreased up to 40 and 20%, respectively (panel B and C), whereas H^5^-*Ss*pCA showed a residual activity of about 60 and 40%, respectively (panel B and C). All these data confirmed that the presence of a thermostable *protein-tag* between the INPN anchoring domain and the *Ss*pCA significantly improved the long-term stability and the storage of this CA.

## 4. Conclusions

The ASL*^tag^* system efficiently overexpressed the chimeric H^5^-*Ss*pCA onto to the bacterial cell surface, as demonstrated by fluorescence microscopy and Western-Blot. As expected, the CO_2_ hydratase assay and the protonography showed that *Ss*pCA was still very active, even linked on the bacterial surface and the H^5^ moiety, showing a CO_2_ hydratase activity similar to that of its anchored counterpart without H^5^. Furthermore, by investigating the behaviour of the whole bacterial cells expressing on the external surface *Ss*pCA or H^5^-*Ss*pCA at different temperatures, we demonstrated an enhancement in terms of thermal stability of the chimeric protein. In conclusion, the H^5^-*Ss*pCA obtained by the ASL*^tag^* system constitutes a valid strategy for further increasing the thermostability of proteins, for processes in which a highly effective, thermostable catalyst is needed.
