# Expression of Concern

**DOI:** 10.1080/14756366.2026.2648252

**Published:** 2026-04-28

We, the Editor and Publisher of *Journal of Enzyme Inhibition and Medicinal Chemistry*, are issuing an Expression of Concern for the following article:

Del Prete, S., Merlo, R., Valenti, A., Mattossovich, R., Rossi, M., Carginale, V., … Capasso, C. (2019). Thermostability enhancement of the α-carbonic anhydrase from *Sulfurihydrogenibium yellowstonense* by using the anchoring-and-self-labelling-*protein-tag* system (ASL*^tag^*). *Journal of Enzyme Inhibition and Medicinal Chemistry*, *34*(1), 946–954. https://doi.org/10.1080/14756366.2019.1605991

After publication, concerns were raised in 2025 by a third party regarding the integrity of Figure 2.

Further investigation by the Journal and Publisher confirmed that the protein bands identified in Lanes 1 and 2 of Figure 2 are duplicates.

The authors have responded to the queries raised, however, given the length of time since publication, the original data for this experiment is unavailable. Therefore, the scientific record could not be corrected.

Whilst the integrity of Figure 2 has not been confirmed, it has been established that Figure 4 supports the findings of Figure 2. The journal confirms there are no other integrity concerns with the article and wishes to notify readers of this and provide reasons why no further action will be taken at this time. However, we advise readers to interpret the information presented in Figure 2 with due caution.

All authors were informed about this Expression of Concern. CC and CTS note their disagreement.

